# Does the type of renal artery anatomic variant determine the diameter of the main vessel supplying a kidney? A study based on CT data with a particular focus on the presence of multiple renal arteries

**DOI:** 10.1007/s00276-017-1930-z

**Published:** 2017-10-05

**Authors:** Marcin Majos, Ludomir Stefańczyk, Zofia Szemraj-Rogucka, Marcin Elgalal, Raffaele De Caro, Veronica Macchi, Michał Polguj

**Affiliations:** 1 Department of Radiology, Barlicki University Hospital, Medical University of Łódź, Kopcińskiego 22, 90-153 Łódź, Poland; 2 Department of Radiological and Isotopic Diagnosis and Therapy, Medical University of Łódź, ul. Pomorska 251, 92-213 Łódź, Poland; 3 Department of Neurosciences, Institute of Human Anatomy, University of Padova, Via A. Gabelli 65, 35127 Padova, Italy; 4 Department of Angiology, Interfaculty Chair of Anatomy and Histology, Medical University of Łódź, Narutowicza 60, 90-136 Łódź, Poland

**Keywords:** Renal artery, Multiple renal arteries, Diameter, Computed tomography angiography

## Abstract

**Background:**

An in-depth knowledge of renal vascular anatomy is essential when planning many surgical procedures; however, a few data exists regarding renal artery diameter. The aim of this study was to assess this morphological feature and to investigate whether a correlation exists between renal artery diameter and the type of arterial supply, with a particular emphasis on variant anatomy and the presence of multiple renal arteries.

**Materials and methods:**

Computed tomography angiography (CTA) studies of 248 patients, i.e., a total of 496 kidneys, were evaluated. The mean age of the patients was 66.4 ± 15.01 years. Renal artery diameter was measured based on the type of arterial blood supply.

**Results:**

The frequency of occurrence of three anatomic variants of renal arterial supply was established: single renal artery (RA) 43.35%, single artery with prehilar branching (pRA) 37.30%, and multiple renal artery (mRA) 19.35%. The diameter of single renal arteries, with either prehilar or hilar branching, was significantly larger than when multiple arteries were present. A detailed analysis of just the mRA variant demonstrated that the diameter of the renal arteries in men was larger (*p* = 0.012) than those in women and that there was no difference in diameter with regard to the side of the body (*p* = 0.219).

**Conclusions:**

The classification described in our study containing a detailed description of renal artery diameter. It may be helpful in clinical practice, especially for transplantologists, surgeons, and vascular surgeons.

## Introduction

An in-depth knowledge of the renal vascular anatomy is of key importance for planning and carrying out both open surgical and endovascular procedures. This is evidenced by the numerous scientific studies in which the morphological features of the renal arteries have been analyzed, either in cadavers or by a variety of imaging techniques such as ultrasonography, classical angiography, computed tomography (CT) or magnetic resonance imaging [3, 5, 9, 10, 16]. These studies have predominantly focused on analyzing variant renal artery anatomy with an emphasis on the presence of accessory arteries [4, 7, 13, 22]. The level of renal artery origin, vessel length, level at which branching occurs, and point of entry into the kidney have all been analyzed as potential parameters.

Such detailed analysis of the morphological features of the renal arterial supply clearly indicates that this is a clinically relevant issue, with regard to numerous pathological conditions. However, no studies have used CT examination to analyze the diameter of the renal artery in detail in a large group of patients. Moreover, this anatomical feature may be essential to fully understanding the process of renal perfusion, as it is not only the number of renal arteries that influences the extent of renal blood supply, but most probably also their diameter. Therefore, the purpose of this study was to investigate renal artery diameter and to look for correlations between this morphological feature and various types of renal arterial blood supply, with a particular emphasis on the incidence of multiple renal arteries. Renal artery diameter was also examined with regard to gender and the side of the body.

## Materials and methods

The study material consisted of 248 patients CT studies (122 women, 126 men) who had undergone computed tomography angiography (CTA) at the Department of Radiology, University Clinical Hospital No. 1, Lodz, Poland. The mean age of the patients was 66.4 years (SD 15.01) and ranged from 24 to 94 years. The median age was 68 years, and the first and third quartiles 58 and 78 years old, respectively. The study was approved by the Bioethics Committee of the Medical University of Lodz, RNN/132/17/KE.

The CT data were acquired from the PACS archiving system of the Department of Radiology, University Clinical Hospital No. 1, Lodz, Poland. It consisted of all the CT studies of patients who successively underwent CTA of an abdominal aorta between January and June 2016.

Criteria for inclusion in the study comprised the presence of two normal kidneys and the absence of morphological features of renal artery disease that could affect the diameter of their lumen (atherosclerosis, arterial dysplasia, arterial wall dissection, thrombosis, etc). The exclusion criteria included nephrectomy (14 patients), renal transplantation (32 patients), and poor quality of CTA study (17 patients).

All CTA studies were performed on a GE Light Speed 64 VCT scanner (GE Healthcare, Milwaukee, WI, USA; 120 kV, 10 mA, dynamic mAbs), 0.625 mm slice thickness, and 0.6 mm pitch; following 80–100 ml intravenous contrast medium administration by an Ultravist 370 (BAYER Schering Pharma AG, Germany), using an automatic injection syringe at a flow rate of 4.5 ml/s. Each study was analyzed on a medical GE Advantage Workstation (AW 4.0, GE Healthcare, Milwaukee, USA).

Each patient study was evaluated in the axial and coronal planes. On the basis of this evaluation, the number of renal arteries was determined for each side and their diameters were measured as accurately as possible, at a distance of 15 mm from their origin in the abdominal aorta.

Quantitative variables were determined by mean, standard deviation (SD), median, minimum, maximum, and quartile. Prior to comparing the values of each of the quantitative variables between any two groups, the normality of the distribution of each of the tested features in these groups was checked using the Shapiro–Wilk test. For normal distributions, the Student’s *t* test was used if the variance was consistent, while the Cochran–Cox test was applied if the variances were not consistent. For non-parametric distributions, the Mann–Whitney *U* test was used.

**Fig. 1:**
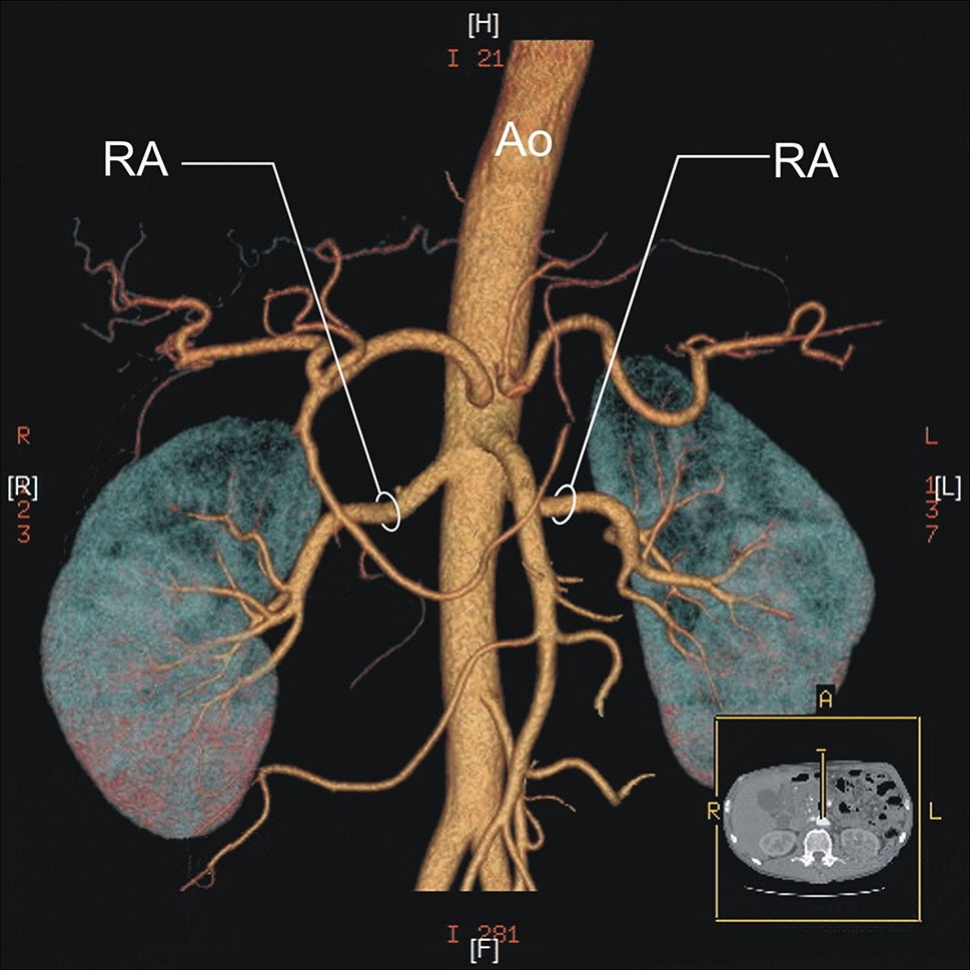
Three-dimensional computed tomographic angiography reconstruction of the abdominal arteries. *Ao* abdominal aorta, *RA* renal artery

**Fig. 2:**
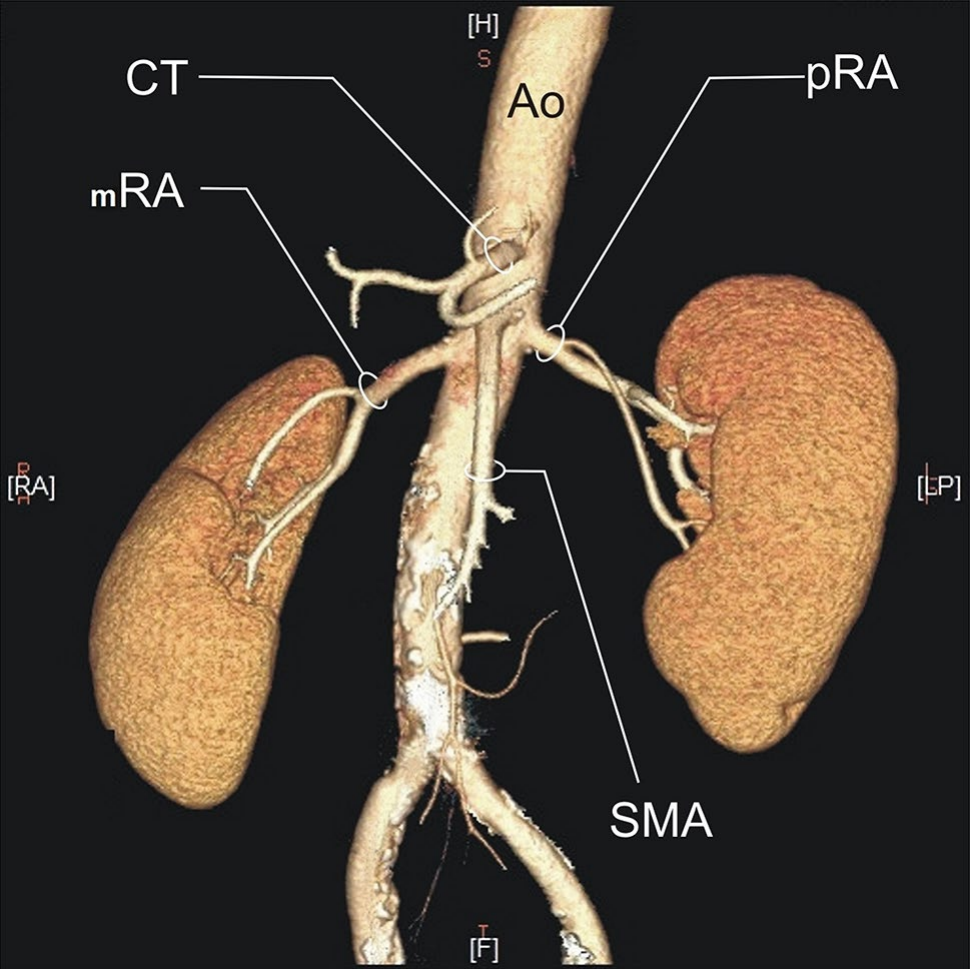
Three-dimensional computed tomographic angiography reconstruction of the abdominal arteries. *Ao* abdominal aorta, *CT* celiac trunk, *pRA* prehilar renal artery, *SMA* superior mesenteric artery

**Fig. 3:**
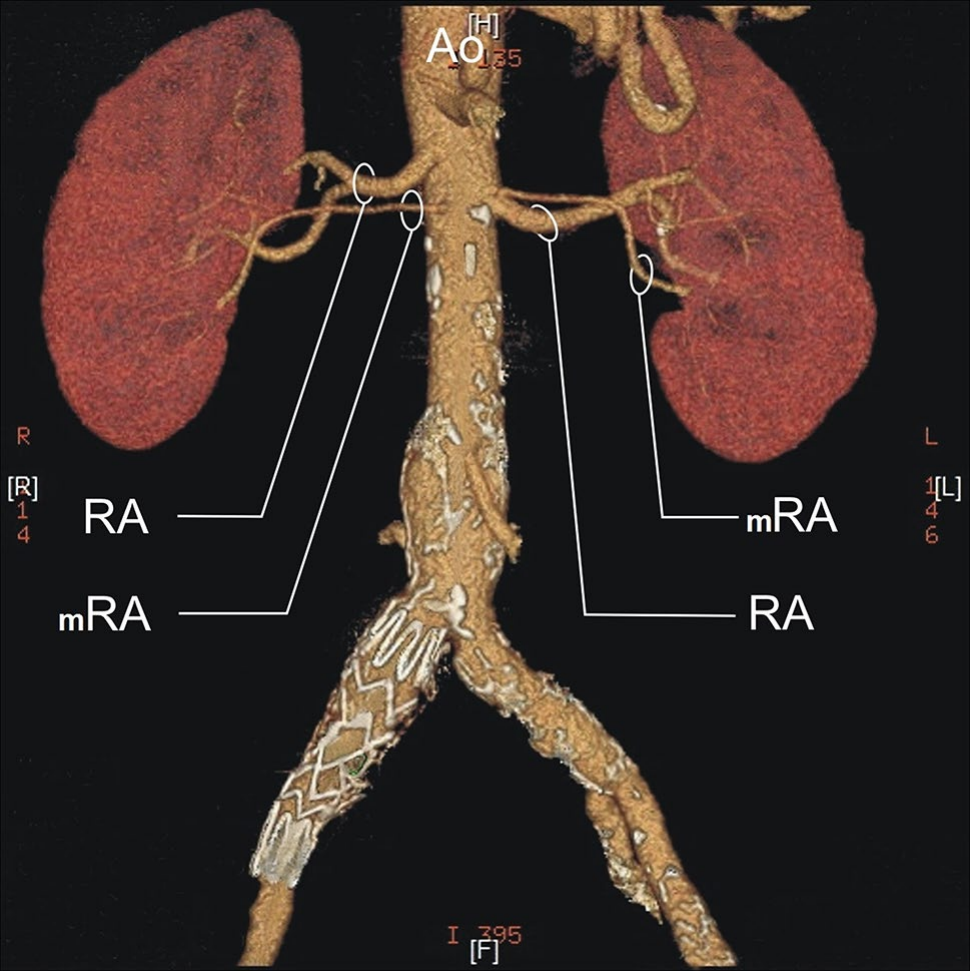
Three-dimensional computed tomographic angiography reconstruction of the abdominal arteries. *Ao* abdominal aorta, *RA* renal artery, *mRA* multiple renal artery

**Fig. 4:**
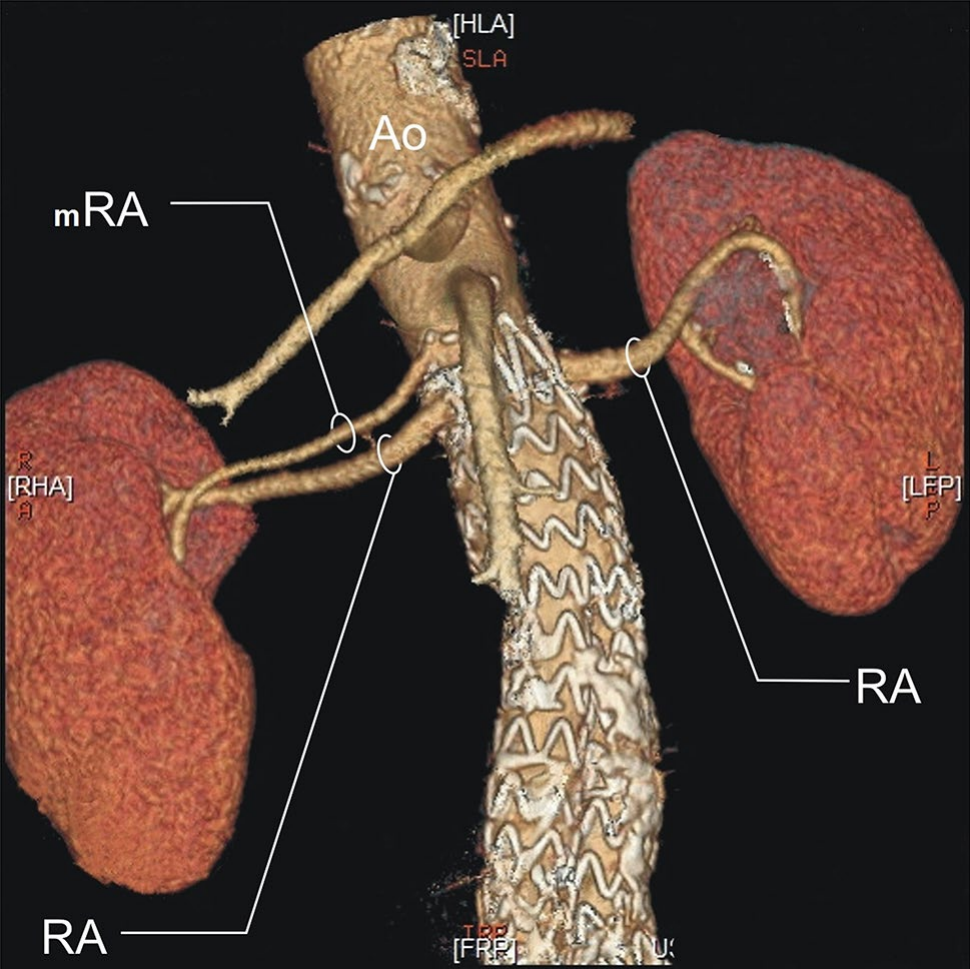
Three-dimensional computed tomographic angiography reconstruction of the abdominal arteries. *Ao* abdominal aorta, *RA* renal artery, *mRA* multiple renal artery

**Fig. 5:**
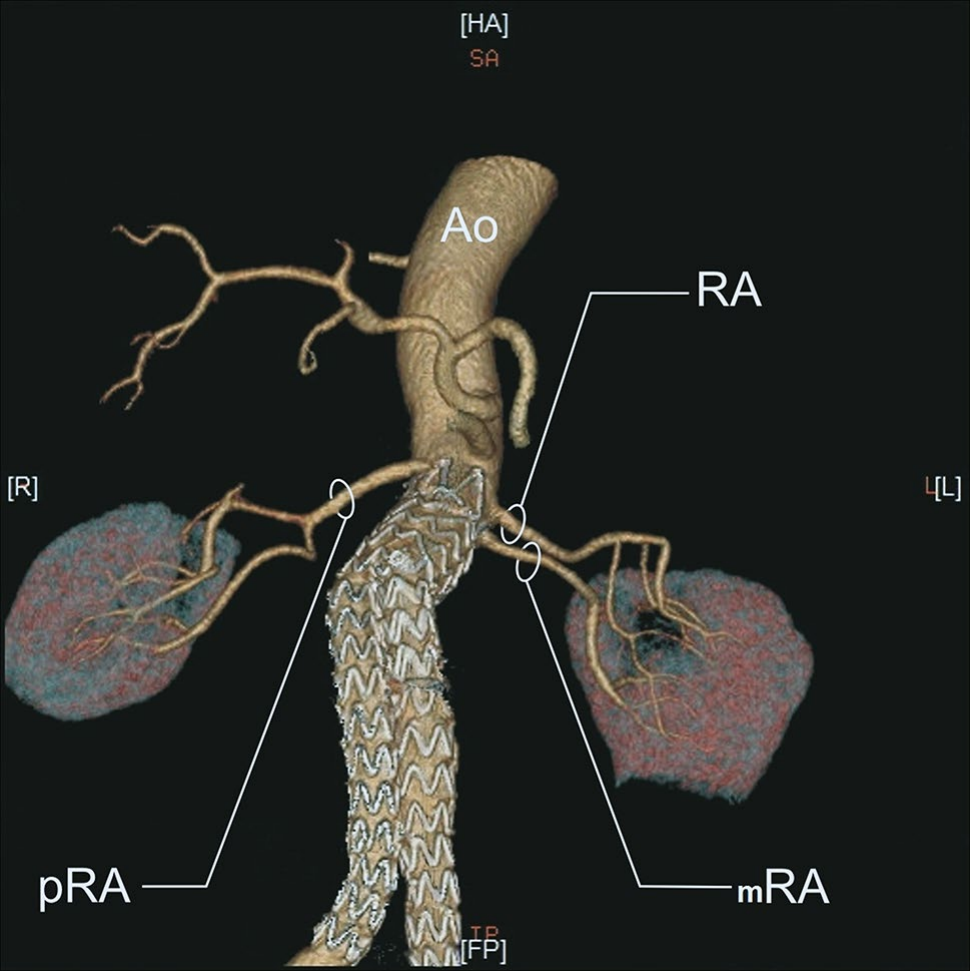
Three-dimensional computed tomographic angiography reconstruction of the abdominal arteries. *Ao* abdominal aorta, *RA* renal artery, *pRA* prehilar renal artery, *mRA* multiple renal artery

To carry out the analysis of renal artery diameter based on the type of arterial blood supply present, the total number of kidneys was established and then divided into different groups according to the following criteria:

1. gender: male, female
2. side: right, left
3. anatomic variant (according to the following classification):

- RA—a single renal artery originating from the aorta and entering the renal hilum (Fig. 1).
- pRA—prehilar renal artery, a single renal artery originating from the aorta and dividing in third proximal of the distance between its origin and the renal hilum, before consequently entering the hilum as two or more separate vessels (first-order branches according to Macchi et al. [11]) (Fig. 2).
- mRA—multiple renal artery, defined as a minimum of two renal arteries originating from the aorta and entering the renal hilum (Figs. 3, 4, 5).

For detailed analysis of the mRA group, i.e., accessory arteries, the total number of patients was taken into consideration (not total no. of kidneys) and analyzed accordingly into the following groups:

A. bilateral accessory arteries (Fig. 3);
B. multiple arteries on the right side only (Fig. 4);
C. multiple arteries on the left side only (Fig. 5);

Subsequently, for each of these groups, patient gender was applied as an additional criterion.

## Results

### Renal artery diameter and anatomic variant of renal blood supply

**Fig. 6:**
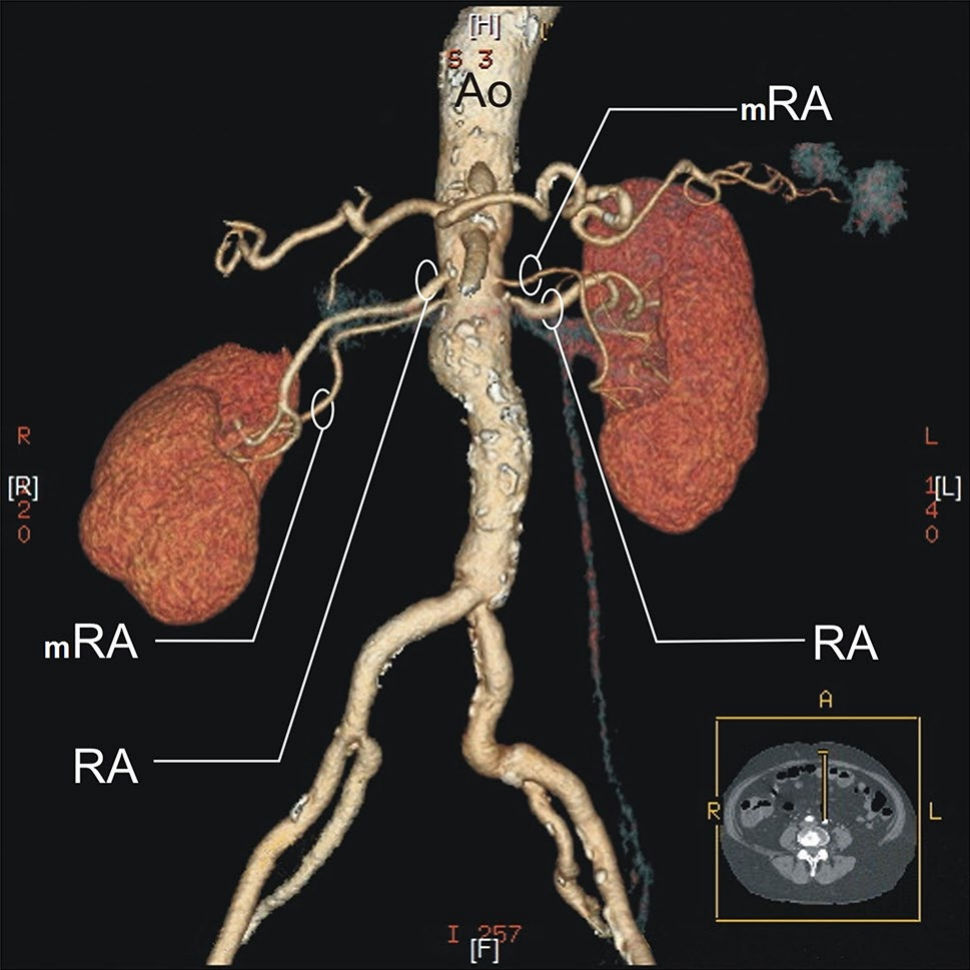
Three-dimensional computed tomographic angiography reconstruction of the abdominal arteries. *Ao* abdominal aorta, *RA* renal artery, *mRA* multiple renal artery

A total of 496 kidneys were analyzed. Anatomic variant type RA was observed in 214 cases (43.35%), pRA in 185 (37.30%), and mRA was observed in 96 cases (19.35%) (Figs. 1, 2, 3, 4, 5, 6). Furthermore, the presence of two renal arteries on one side (1RA and one or more mRA) was observed in 90 cases and three arteries in six cases (Figs. 3, 6). For the female group, RA was observed in 130 (53.28%), pRA in 77 (31.56%), and mRA in 37 (15.16%), while in the male group, RA in 84 (33.33%), pRA in 109 (43.25%), and mRA in 59 (23.41%). The spread ranged from 2.4 to 9.8 mm for the RA variant, 3.0–9.7 mm for pRA and between 2.4 and 9.8 mm for both single artery variants (i.e. RA + pRA); for multiple arteries, the spread ranged from 3.3 to 9.3 mm. Detailed data describing the renal artery diameters of the entire study group are shown in Table 1.

**Table 1:** Statistical analysis of renal artery diameter values for the study group

Anatomic variant	*N*			Average diameter (mm)			SD			Median (mm)		
	♀	♂	*R*	♀	♂	*R*	♀	♂	*R*	♀	♂	*R*
RA	130	84	214	5.90	6.34	6.01	1.1	1.3	1.2	6.0	6.4	6.2
pRA	77	109	185	5.75	6.46	6.17	1.2	1.3	1.3	5.7	6.4	6.1
RA + pRA	207	192	399	5.85	6.41	6.12	1.1	1.3	1.2	5.8	6.4	1.2
mRA	37	59	96	5.17	5.77	5.54	1.0	1.2	1.2	5.1	5.7	5.5

*RA* single renal artery originating from the aorta and entering the renal hilum, *pRA* single renal artery originating from the aorta and branching proximal to the renal hilum and entering it as two or more branches, *mRA* a min. of two renal arteries originating from the aorta and entering the renal hilum

Statistically significant differences in renal artery diameter values were found between the single artery variant group (RA) and multiple artery variant group (mRA)—*p* < 0.001, between the prehilar variant group (pRA) and the multiple artery variant group (mRA)—*p* < 0.001, and between both single renal artery variants (RA + pRA) and the multiple renal artery group mRA—*p* < 0.001, Table 1.

### Renal artery diameter and anatomic variant of renal blood supply depending on patient gender

**Fig. 7:**
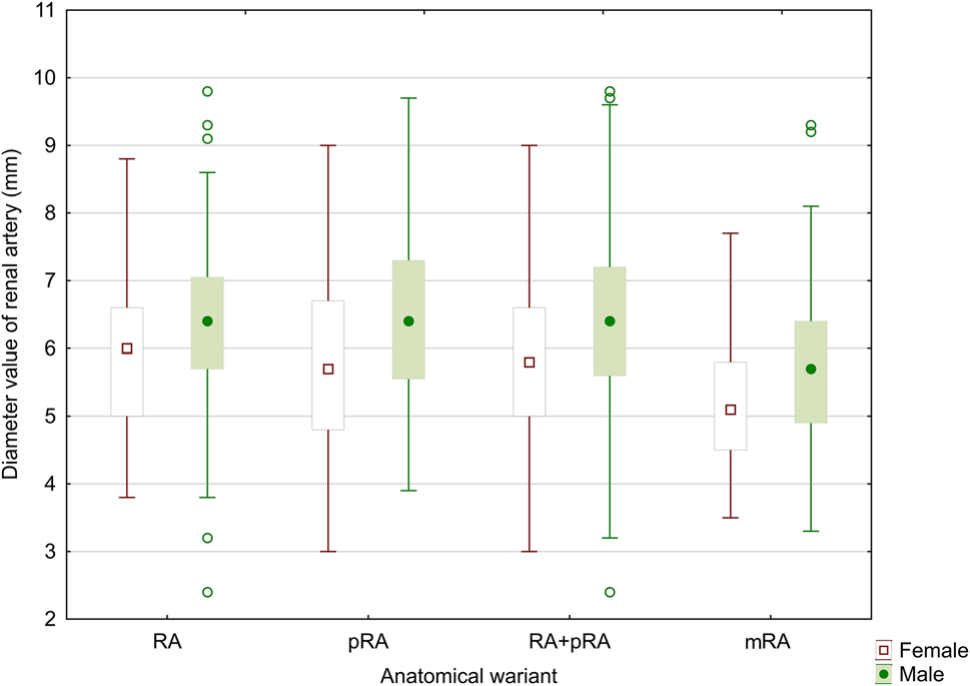
Statistical results of renal artery diameter values for the male and female groups

Detailed data describing the renal artery diameter values for the female group and for the male group are given in Table 1 and Fig. 7. In the female group, the RA variant spread ranged from 3.8 to 8.8 mm, for pRA from 3.0 to 9.0 mm, both single artery variants (RA + pRA) from 3.0 to 9.0 mm, and for multiple arteries 3.5 to 7.7 mm. For men, these ranges ranged from 2.4 to 9.8 mm, 3.9 to 9.7 mm, 2.4 to 9.8 mm, and 3.3 to 9.3 mm, respectively.

Statistically significant differences in renal artery diameter values were observed between men and women in the RA group, (*p* = 0.005), in the pRA group (*p* < 0.001), and in the RA + pRA group (*p* < 0.001), as well as in the mRA group (*p* = 0.012).

In the right-sided group, the renal artery diameter was significantly larger in men for the pRA variant and mRA variant, with *p* values of *p* < 0.001 and *p* = 0.002, respectively. However, in the left-sided group, statistically significant differences in men were only observed for the RA variant (*p* = 0.014) (Table 2).

**Table 2:** Statistical differences in renal artery diameters between men and women depending on the side of the body

Anatomic variant	Renal arteries on both right side and left side (*p*)	Renal arteries on right side (*p*)	Renal arteries on left side (*p*)
RA	0.006	0.221	0.014
pRA	< 0.001	< 0.001	0.163
RA + pRA	< 0.001	< 0.001	0.005
mRA	0.012	0.002	0.537

*RA* single renal artery originating from the aorta and entering the renal hilum, *pRA* single renal artery originating from the aorta and branching proximal to the renal hilum and entering it as two or more branches, *mRA* a min. of two renal arteries originating from the aorta and entering the renal hilum

### Renal artery diameter and anatomic variation of renal blood supply depending on side of the body

No significant relationship was found between the renal artery diameter and the side of the body for each type of variant blood supply. Furthermore, while there were no such differences in the male group, significantly larger renal artery diameters for pRA and mRA were observed in the female group (Table 3).

**Table 3:** Statistical differences in renal artery diameters depending on the side of the body in the male and female groups

Anatomic variant	Women (*p*)	Men (*p*)	Together (*p*)
RA	0.965	0.475	0.645
pRA	0.039	0.965	0.127
RA + pRA	0.115	0.990	0.241
mRA	0.015	0.726	0.220

*RA* single renal artery originating from the aorta and entering the renal hilum, *pRA* single renal artery originating from the aorta and branching proximal to the renal hilum and entering it as two or more branches, *mRA* a min. of two renal arteries originating from the aorta and entering the renal hilum

### Diameter of the dominant accessory renal arteries depending on gender and side

**Fig. 8:**
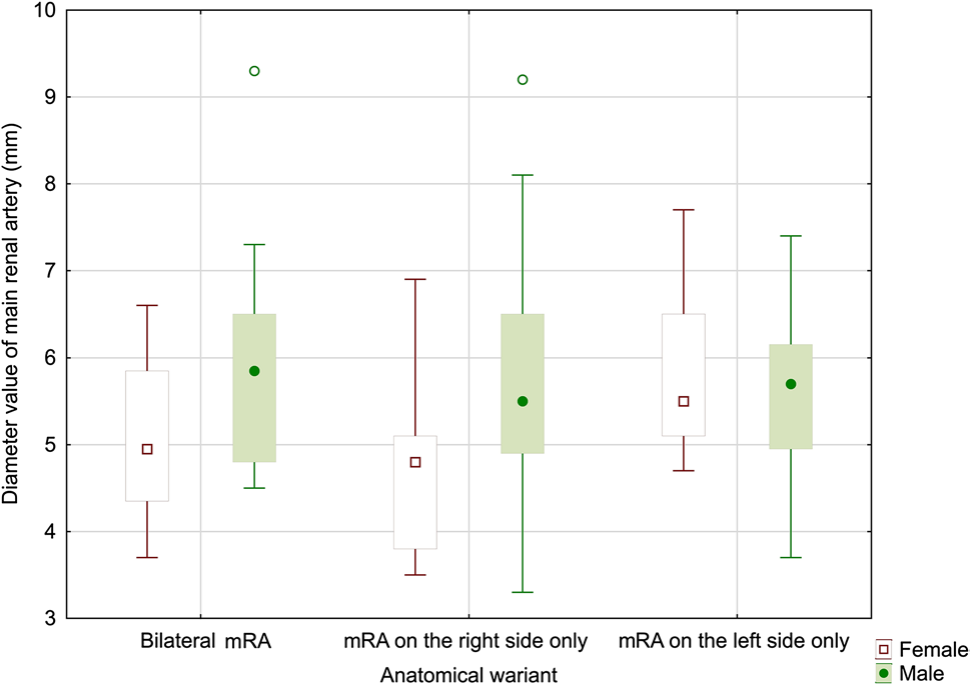
Statistical results of dominant multiple artery diameter values for the male and female groups

Detailed data describing the diameters of dominant accessory renal arteries for the entire study group, including the unilateral or bilateral incidence of these arteries, are given in Table 4 and Fig. 8. In the female group, the diameter values ranged between 3.7 and 6.6 mm for bilateral accessory arteries, between 3.5 and 6.9 mm for unilateral right-sided accessory arteries, and 4.7 to 7.7 mm for the left-sided unilateral accessory arteries. In the male group, these values ranged from 4.5 to 9.3 mm, 3.3 to 9.2 mm, and 3.7 to 7.4 mm, respectively.

**Table 4:** Statistical analysis of the dominant accessory renal artery diameter values

Anatomic variant	*N*			Average diameter (mm)			SD			Median (mm)		
	♀	♂	*R*	♀	♂	*R*	♀	♂	*R*	♀	♂	*R*
Bilateral	16	18	34	5.06	5.93	5.52	1.0	1.2	1.16	4.9	5.9	5.6
Unilateral	10	25	35	4.66	5.80	5.47	1.0	1.4	1.40	4.8	5.5	5.1
Only left side	11	16	27	5.80	5.54	5.64	0.9	0.9	0.93	5.5	5.7	5.7

For the entire study group, no statistically significant differences were found between the right and left sides with regard to the diameter of the dominant renal arteries, for both the bilateral (*p* = 0.581) and unilateral (*p* = 0.256) incidences of these arteries.

Statistically significant differences in dominant accessory renal artery diameter values between women and men were observed for bilateral incidence (*p* = 0.034) and for unilateral right side only accessory arteries (*p* = 0.027). However, no significant dependence was observed for unilateral left side only accessory arteries (*p* = 0.483). Moreover, no statistically significant differences were found between the right and left sides in the female and male groups with bilateral accessory renal arteries. When accessory arteries were present on one side only, statistically significant differences were observed for the left renal artery in the female group (*p* = 0.015); however, no statistically significant differences were seen in the male group.

## Discussion

Since the beginning of the modern era, the numerous anatomic variants of the renal blood supply and the clinical relevance of the kidney vasculature have served as the motivation for numerous researchers to find a reliable method of visualizing the renal arteries [21]. For this purpose, both autopsy and imaging techniques have been used: ultrasound, CTA, and magnetic resonance angiography [1, 2, 12, 14]. Presently, based on the literature and the personal experience of the authors, CT is arguably the method with the highest diagnostic value with regard to morphological evaluation of the renal arteries, primarily due to its high spatial resolution [6, 8, 10, 17, 18]. Consequently, to evaluate renal artery diameter, a group of patients that had undergone CTA for various reasons was selected and then divided into three groups comprising different variants of the renal arterial supply: RA, pRA, and mRA.

The study material was divided into two groups consisting of subjects with either one single major artery or more than one renal artery, and the single renal artery group was then further divided into two groups, i.e., RA variant and pRA, resulting in three variants in total. These two subgroups (RA variant and pRA variant) were classified on the basis of the level at which the single renal artery divided. In the RA variant, branching of the main artery into segmental arteries occurred within the pelvis of the kidney, whilst for the pRA variant, it occurred in third proximal of the distance between its origin and the renal hilum. These subtypes were commonly present in the CTA images examined in the present study. In addition, to compare the results of this study with data from the literature, not only the three aforementioned subtypes were analyzed, but also the total number of kidneys that were supplied by a single artery, i.e., the sum of the RA and pRA subtypes, were compared to kidneys supplied by more than one renal artery—mRA subtype.

In the material evaluated, it was observed that the diameter of single renal arteries, i.e., both the RA and prehilar variants, was significantly larger than that of the dominant accessory renal artery in the multiple artery variant (*p* < 0.001). Understandably, the most significant statistical difference was found between the sum of all kidneys supplied by a single renal artery, i.e., RA + pRA and mRA due to the size of these groups. Furthermore, Ramadan et al. [19] have proposed a formula that estimates, with a high level of probability, the incidence of accessory arteries based on the dominant renal artery diameter, and the length of the kidney that it supplies [19].

An analysis of the renal artery diameters across the entire study group found that the values for men were significantly higher than for women for each type of renal artery variant. This correlation is most probably due to the fact that most anthropometric parameters are generally larger in men than in women. On the other hand, the most significant statistical difference between the gender groups was observed for the pRA variant, and this may be due to the difference in quantitative distribution of the different types of renal artery variants between women and men: for women, the most common type was RA (53.28%), whilst for men, it was pRA (43.25%).

The sizes of the left and right renal arteries were also analyzed in relation to gender. Interestingly, in the male group, while larger diameters were observed for the pRA and mRA variants in the right kidney group, only the RA variant was larger in the left kidney group. This arguably indicates that the side of the body on which the kidney is located does not influence the diameter of the renal artery.

The mean value of RA measured in the present study was 6.1 mm, values that differ from data of a previous study of our group (4 ± 0.83 mm) [11]. These differences can be ascribed to the analyzed point of evaluation of the diameter at a distance of 15 mm from their origin in the abdominal aorta in the present study and at the level of origin in the study of Macchi et al. [11] and to the different studied material (living patient versus corrosion cast).

The results obtained in this study differ from those published by Satyapal et al. [20] in a study based on a similar amount of material consisting of 130 angiography studies and 106 autopsies in South Africa. No statistically significant differences were found in renal artery size between men and women; however, it was shown that the average renal artery diameter for women was 4.3 mm and for men 5.1 mm. This lack of statistical significance was most likely related to the heterogeneity of the population evaluated, for which the SD was 1.7 mm for women and 2.1 mm for men; in our study, these respective differences were 1.1 and 1.3 mm. Furthermore, different research techniques were used in our study and in that of Satyapal et al. [20]. During an autopsy, there are considerable technical barriers that make the process of visualizing small arteries very difficult and may result in damage to the vessels, which can potentially impair any results obtained. In contrast, the CTA approach used in the present study is considered to be the “gold standard” for imaging of renal arteries.

Furthermore, when comparing the mean renal artery diameters between the study by Satyapal et al. [20], and the results of this study, it was observed that in our material, the mean renal artery diameter values are 1.55 mm higher for women and 1.31 mm for men. These differences may be due to the disparate research techniques used to acquire the measurements and/or the differences in the populations evaluated.

The present study also analyzed the relationship between of renal artery diameter and the side of the body on which the kidney was located, as establishing whether such a correlation exists is an important factor from the point of view of clinical practice. It is a well-known fact that the location of the kidney plays an important role when making decisions regarding its transplantation. A study by Özdemir-van Brunschot et al. [15] on an impressive amount of material consisting of 9718 patients confirmed that transplantation of the left kidney results in significantly fewer complications in recipients compared with transplantation of the right kidney. This is clearly related to the difference between the length of the right and left renal veins and also to the relationship between the length of the left renal veins and arteries. With this in mind, it is reasonable to assume that the diameter of the renal arteries may also be an important factor in the anatomico-topographic correlations of renal vessels. However, no statistically significant differences were found between the right and left kidneys with regard to renal artery diameter. Despite this, the left renal artery was found to be significantly wider than the corresponding artery on the right side for the pRA and mRA variants in women. On the other hand, no such correlation was found in the whole study group or in the male group with regard to the RA variant.

According to our observations when comparing renal artery diameters between men and women, it was observed that the diameters of the dominant accessory arteries in the male group were significantly larger for both the bilateral and unilateral right-sided accessory arteries. Whereas for unilateral left-sided only accessory arteries, the average renal artery diameter was slightly lower than in women, on average by 0.3 mm.

No significant correlation was observed between the dominant renal artery diameter and the side of the body for the whole study group, nor for the male group. The only exception was the presence of a significant correlation between renal artery diameters for unilateral accessory arteries in women, in which the dominant accessory artery on the left was found to be wider than on the right.

It should be noted that the above evaluation in this study was limited to a certain degree by the relatively small number of mRA variants. This issue will be addressed in the subsequent studies.

This study is the detailed assessment of renal artery diameter in this context. However, the presented data are intended as only a part of a broader project in which a complete, multi-faceted assessment of the renal vasculature will be carried out. This project aims to address real clinical needs, for which an in-depth knowledge of the vastly diverse renal vascular anatomy is crucial for therapeutic decision making, in particular with regard to the treatment of significant pathologies such as renal hypertension or abdominal aortic aneurysms, as well as when planning renal transplant procedures.

## Conclusions

The increasing number of interventions at the renal region underlines the importance of knowledge describing the diameter of renal arteries. The classification described in our study represents a detailed description of renal artery diameter; such information may be especially helpful for transplantologists, surgeons, and vascular surgeons.
